# Perillyl alcohol has antibacterial effects and reduces ROS production in macrophages

**DOI:** 10.1590/1678-7757-2019-0519

**Published:** 2020-04-27

**Authors:** Rebeca Dantas Alves FIGUEIREDO, Adriana Cabrera ORTEGA, Laura Andrea GONZÁLEZ MALDONADO, Ricardo Dias de CASTRO, Mario Julio ÁVILA-CAMPOS, Carlos ROSSA, Sabrina Garcia de AQUINO

**Affiliations:** 1 Universidade Federal da Paraíba Programa de Pós-Graduação em Odontologia João PessoaParaíba Brasil Universidade Federal da Paraíba, Programa de Pós-Graduação em Odontologia, João Pessoa, Paraíba, Brasil.; 2 Universidade Estadual Paulista Faculdade de Odontologia de Araraquara Departamento de Diagnóstico e Cirurgia AraraquaraSão Paulo Brasil Universidade Estadual Paulista (UNESP), Faculdade de Odontologia de Araraquara, Araraquara, Departamento de Diagnóstico e Cirurgia, São Paulo, Brasil.; 3 Universidade de São Paulo Instituto de Ciências Biológicas Departamento de Microbiologia São Paulo Brasil Universidade de São Paulo, Instituto de Ciências Biológicas, Departamento de Microbiologia, São Paulo, Brasil.

**Keywords:** Periodontal diseases, Immunomodulation, Macrophages, Natural products

## Abstract

Natural products have emerged as a rich source of bioactive compounds for adjunctive treatments of many infectious and inflammatory conditions, including periodontitis. Among the monoterpenes with significant biological properties, there is the perillyl alcohol (POH), which can be found in several essential oils and has shown immunomodulatory properties in recent studies, which may be interesting in the treatment of non-neoplastic inflammatory disorders.

**Objective:**

To determine the antibacterial and immune modulatory activities of the POH.

**Methodology:**

The minimum inhibitory concentration (MIC) and the minimum bactericidal concentration (MBC) of the POH for two significant Gram-negative periodontal pathogens were determined by macrodilution and subculture, respectively. Cell proliferation and cytotoxicity in RAW 264.7 macrophages were determined by Trypan Blue and mitochondrial enzymatic activity assay. The modulation of reactive oxygen species (ROS) was analyzed by flow cytometry and expression of TNF and arginase-1 by real-time PCR.

**Results:**

The POH was effective against *P. gingivalis* (ATCC 33277) and *F. nucleatum* (ATCC 25586) with MIC= MBC=1600 μM. No cytotoxicity up to 100 µM was observed on macrophages. The cell proliferation was inhibited from 48 hours at 100 μM (p<0.05) and 250 μM (p<0.01). The POH increased ROS production at both 10 μM and 100 μM (p<0.05) in unstimulated cells. The PMA-induced ROS production was not affected by POH, whereas 100 μM significantly reduced lipopolysaccharide-induced (LPS-induced) ROS. The expression of TNF was not affected by POH in unstimulated cells or in cells polarized to M1 phenotype, whereas both concentrations of POH reduced (p<0.05) the expression of arginase-1 in M2-polarized macrophages.

**Conclusion:**

The POH has antibacterial activity against periodontal pathogens and reduced proliferation of murine macrophages without significant cytotoxicity at concentrations up to 100 μM. In addition, the POH reduced the LPS-induced ROS and the expression of arginase-1 in M2-polarized macrophages.

## Introduction

In the last decades, advances in periodontal studies have increased the knowledge about the pathogenesis and its immunological mechanisms that modulate the host response to microbial challenge. Thus, although the onset of periodontitis is dependent on the maturation of a complex microbial biofilm (essentially composed of Gram-negative anaerobes), the progression, the extent, and the severity of the disease are dependent on the balance between pro-inflammatory and protective mediators, which involves complex host-microbial interactions.^1^

In this context, some cell types are particularly significant for tissue homeostasis in the periodontal microenvironment, such as macrophages. The macrophages are highly prevalent in diseased periodontal sites, and as the prototypical antigen-presenting cell, are directly involved in the response to the microbial challenge.^2^ Activated macrophages also secrete important mediators in extracellular matrix degradation and resorption of alveolar bone, and can vary into bone-resorbing osteoclasts.^3 - 5^

Depending on the external cues present in inflammatory microenvironment, the macrophages can assume different phenotypes in a spectrum between the extremes of the classically activated or pro-inflammatory (M1), and alternatively activated or reparative (M2) macrophages, according to the major cytokines secreted. The characterization of macrophage response and phenotype associated with inflammatory conditions, including periodontal diseases, may be useful in developing new treatment strategies.^6^

There has been a growing interest in natural products with antimicrobial and anti-inflammatory properties as adjunct treatments for many infectious and inflammatory conditions, including periodontitis.^7 - 9^ These products include essential oils, which are volatile aromatic liquids extracted from plants and are active ingredients of phytotherapy.^9^ Monoterpenes are the main constituents of essential oils, and most studies of their effects on human health have been performed with limonene, carvone, camphor, and perillyl alcohol (POH). The POH can be found in several essential oils,^10 - 12^ and medical interest in this compound was initially based on its anti-tumoral activity.^13 - 17^

Recent studies have shown immunomodulatory properties of the POH, which may be of interest in the treatment of non-neoplastic inflammatory disorders. D’Alessio, et al.^18^ (2014) reported that the POH promotes wound healing, likely by decreasing the pro-inflammatory cytokines IL-6 and TNF. The POH reduced oxidative activity, TNF production, and NF-kB activation in a rat model of ethanol-induced acute hepatic injury.^19^ In addition, the antinociceptive activity of the POH has recently been shown in a murine model of orofacial pain.^20^

Despite evidence of the immunomodulatory potential of the POH, few studies have assessed the biological mechanisms of the POH on cells directly involved in the pathogenesis of inflammatory disorders, including periodontitis. Considering the fundamental roles of the Gram-negative microorganisms and of the macrophages in the etiopathogenesis of periodontal diseases, this study determines the antimicrobial activity and the POH effects in the macrophage response.

## Methodology

### Determination of antimicrobial activity against periodontal pathogens

The antimicrobial susceptibility test was performed according to the broth macrodilution method, M11-A5, of the CLSI (Clinical and Laboratory Standards Institute) with some modifications.^21^ The bacterial inoculum was prepared by the suspension of colonies of *Fusobacterium nucleatum* (ATCC 25586, Manassas, VA, USA) and *Porphyromonas gingivalis* (ATCC 33277, Manassas, VA, USA) in 5 mL of brain heart infusion (BHI) broth supplemented with hemin (5 μg/mL) and menadione (1 μg/mL of vitamin K), followed by incubation under anaerobic conditions (90% N_2_ and 10% CO_2_) at 37°C for 48 h. Then, the inoculum was adjusted to 1.5x10^8^ colony forming units (CFU)/mL. The POH stock solution (50 mg/mL) was prepared using 4% dimethyl sulfoxide as the solvent (DMSO, Sigma-Aldrich, St. Louis, MO, USA). Increasing POH concentrations (0.00781-1 mg/mL) were used for testing, and 0.12% chlorhexidine gluconate (Periogard^®^; Colgate-Palmolive Ind. Bras., Osasco, SP, Brazil) was used as a positive control. The minimum inhibitory concentration (MIC) was determined by observing the mean turbidity and/or presence of sediment. To determine the minimum bactericidal concentration (MBC), 10 μL aliquots from the test tubes without visible growth were plated on blood agar supplemented with hemin (5 μg/mL) and menadione (1 μg/mL of vitamin K), and then incubated under anaerobic conditions (90% N_2_ and 10% CO_2_) at 37°C for ٧٢ h. Three independent experiments assessed in triplicate were performed.

### Cell line and preparation of perillyl alcohol

The commercially available RAW 264.7 murine macrophage cell line (ATCC #TIB-71) was used in this study. This cell line has been widely used in the literature and is responsive to LPS, PMA, and polarization induced by LPS and IFN-γ (M1) or IL-4 (M2).^22 - 24^

The cells were cultured in Dulbecco’s Modified Eagle Medium (DMEM) supplemented with 10% heat inactivated fetal bovine serum (FBS), 100 U/mL penicillin, and 100 µg/mL streptomycin and then maintained in a humidified atmosphere at 37ºC containing 5% CO_2_. The (S)-(-)-perillyl alcohol, 96%, was obtained from Sigma Aldrich Chemical Co (St. Louis, MO, USA). A stock solution (25 mM) was prepared in FBS and diluted in DMEM at the final concentrations of 10 µM, 25 µM, 50 µM, 100 µM, and 250 µM for the experiments.

### Cytotoxicity assays: trypan blue and MTS assay

Cytotoxicity was assessed by the trypan blue dye exclusion test and by the mitochondrial enzymatic activity (MTS) assay (3-[4,5,dimethylthiazol-2-yl]-5-[3-carboxymethoxy-phenyl]-2-[4-sulfophenyl]-2H-tetrazolium). The RAW 264.7 cells were plated in 96-well plates at densities of 1x10^5^and 5x10^4^cells/well for the trypan blue test and MTS assay, respectively, and stimulated with POH at concentrations of 10 µM, 25 µM, 50 µM, 100 µM, and 250 µM for 24 h. The negative control consisted of non-stimulated cells, and the positive control consisted of cells treated with 6 µM camptothecin (Sigma-Aldrich Co, St. Louis, MO, USA). After the experimental period, the cells were resuspended at a ratio of 1:1 with 0.4% trypan blue (Gibco, Thermo Fisher Scientific, Waltham, MA, USA) and counted in a hemocytometer after 2 minutes of incubation. The results were expressed as percentage of viable cells regarding the total number of cells.

The MTS assay determines the number of viable cells using the activity of mitochondrial dehydrogenases that convert tetrazolium salts to formazan, generating a colorimetric reaction.^25 , 26^ The MTS assay was conducted according to the manufacturer’s instructions (CellTiter 96^®^ AQueous One Solution Cell Proliferation Assay, Promega, Madison, WI, USA). The absorbance was measured at 490 nm in a plate reader (Spectramax L, Molecular Devices, Sunnyvale, CA), and the enzyme activity in the treatment groups was estimated as a percentage regarding the respective negative controls.

### Cell proliferation assay

The RAW 264.7 cells were plated in 96-well plates at a density of 1x10^4^ cells/well and stimulated with POH at concentrations of 10 µM, 25 µM, 50 µM, 100 µM, and 250 µM for periods of 24, 48, and 72 h. The negative control consisted of the non-stimulated cells, and 10 µg/mL mitomycin was used as the positive control (Sigma-Aldrich Co, St. Louis, MO, USA). At each experimental period, the cells in suspension in the culture medium and the attached cells (removed by a 5-minute incubation in 0.25% trypsin) were resuspended at a 1:1 ratio with 0.4% trypan blue, then incubated for 2 minutes at room temperature, and the number of viable (unstained) and dead (blue) cells were estimated in a hemocytometer by a trained examiner. The results were expressed as the absolute number of live cells.

### Production of reactive oxygen species (ROS)

The POH effect on ROS production was assessed by flow cytometry. Macrophages were seeded in 6-well plates (3x10^5^ cells *per* well). After 24 h of incubation, the culture medium was removed, the cells were gently washed with phosphate buffered saline (PBS) twice (pH 7.4, without Ca and Mg), and PBS containing 10 µM 6-carboxy-2’,7’-dichlorodihydrofluorescein diacetate (cat# C400, Molecular Probes, Thermo Fisher Scientific, Waltham, MA, USA). The cells were loaded with this compound (a molecular ROS sensor) by incubation at 37°C for 30 minutes. After the removal of the reagent and washing the cells with PBS, the POH was added at 10 µM and 100 µM in complete culture medium. After 30 minutes, the cells were stimulated with 50 ng/mL phorbol myristate acetate (PMA) (cat# P1585, Sigma-Aldrich Co, St. Louis, MO, USA) and 10 µg/mL LPS ( *E. coli* , serotype 0111:B4, cat# L4130, Sigma-Aldrich Co, St. Louis, MO, USA) for 20 minutes to induce ROS production. The cells were harvested by trypsinization, resuspended in PBS containing 2% FBS, and analyzed by flow cytometry (BD FacsVerse, BD Biosciences, San Jose, CA, USA). A minimum of 10,000 events were acquired and the percentage of positive events in the FL1 (FITC) channel (ROS-producing cells) was recorded. Three independent experiments were performed. Data are expressed as the percentage of cells emitting fluorescence in the FL1 channel (ROS-positive cells).

### TNF and arginase gene expression

The POH effect on expression of M1/M2-related genes was determined by analyzing TNF (M1) and Arginase-1 (M2) by reverse transcriptase real-time PCR (RT-qPCR). Macrophages were plated in 6-well plates (3x10^5^ cells/well). After a 4 h incubation to allow cell adhesion, the plates were treated with POH at 10 µM and 100 µM for 24 h. After 24 h, the macrophages were stimulated with LPS (1 µg/mL, Sigma Aldrich Co, St. Louis, MO, USA) and recombinant murine IFN-γ (100 ng/mL, cat# 315-05, Peprotech Inc, Rocky Hill, NJ, USA) as the M1-polarizing conditions, or with recombinant murine IL-4 (40 ng/mL, cat# 214-14, Peprotech Inc, Rocky Hill, NJ, USA) as the M2-polarizing condition. After an additional 24 h, the adhered cells were collected, and the total RNA was isolated using an affinity column system including treatment with DNAse (Total RNA Purification Kit, cat# DPK-108, Cellco Biotec, São Carlos, SP, Brazil). The concentration and purity of the RNA were determined in a UV spectrophotometer, and 300 ng of total RNA was used for synthesis of complementary DNA (cDNA) using random hexameters as primers and recombinant transcriptase, according to the instructions of the supplier of the reagents (High capacity cDNA reverse transcription, cat# 4368814, Applied Biosystems, Thermo Fisher Scientific, Waltham, MA, USA). The cDNA was used in qPCR reactions using the TaqMan chemistry (TaqMan Fast Advanced, cat# 4444556, Applied Biosystems, Thermo Fisher Scientific, Waltham, MA, USA) and the pre-designed and optimized sets of primers and probe for each gene of interest (TaqMan gene expression assays, cat# 4331182, Applied Biosystems, Thermo Fisher Scientific, Waltham, MA, USA) on a StepOne Plus qPCR thermocycler (Applied Biosystems, Thermo Fisher Scientific, Waltham, MA, USA). The GAPDH was used as the internal control, and relative gene expression was determined using the Δ(ΔCt) method.

### Statistical analysis

Before the analysis, the data normality was verified with the Shapiro-Wilk test. For comparisons between the experimental groups, the ANOVA followed by the Tukey’s *post hoc* test were used. Data are expressed as the mean ± standard deviation, and the analysis were performed in the GraphPad version 7.0 with the significance level set at 95% (p<0.05).

## Results

### Antimicrobial activity of the POH against periodontal pathogens

The POH had an antimicrobial activity against *P. gingivalis* and *F. nucleatum* at the same concentration (MIC 1600μM), and the MBC was equal to the MIC for both microorganisms ( Table 1 ).

**Table 1:** Minimum inhibitory concentration (MIC) and minimum bactericidal concentration (MBC) given in μg/mL for POH and chlorhexidine at 0.12% (control) against strict anaerobes

	*P. gingivalis*		*F. nucleatum*	
Substances	MIC (μM)	MBC (μM)	MIC (μM)	MBC(μM)
POH	1600	1600	1600	1600
Chlorhexidine	0.0000037	0.0000037	0.00000742	0.00000742

### Cytotoxicity and cell proliferation assays

**Figure 1:**
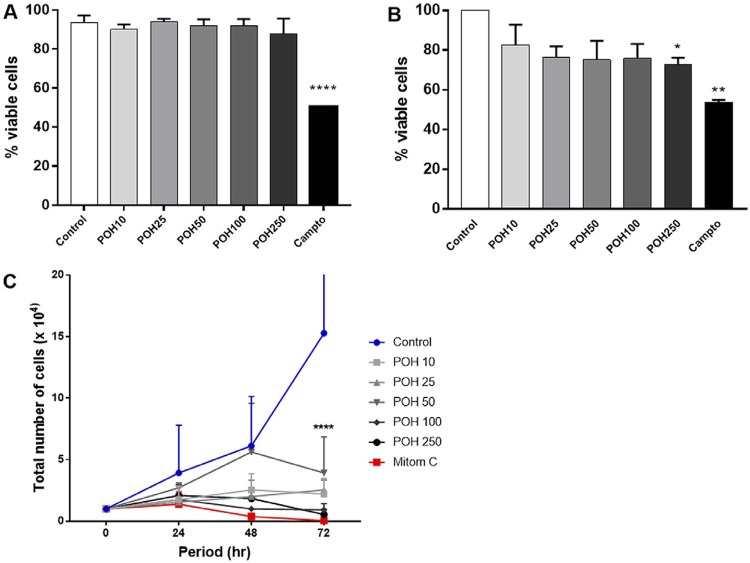
POH effect on macrophage viability and proliferation. The RAW 264.7 cells were treated with POH (10-250 µM) and cultured *in vitro* for 24, 48, or 72 h. The viability was determined by the trypan blue (A) and MTS (B) assays at 24 h. The bars represent the means, and the vertical lines represent the standard deviations of three independent experiments. C): POH effect on proliferation of RAW 264.7 cells at 24, 48, and 72 h. Each horizontal line represents an experimental condition, and the vertical lines represent the standard deviations of three independent experiments. The asterisks (****) indicates a significant difference compared to the negative control (2-way ANOVA, p<0.0001)

The RAW 264.7 cells were treated with the indicated concentrations of POH (10-250 µM) for 24 h. Cell viability was not significantly affected by up to 250 µM of POH, as determined by the trypan blue exclusion test (over 85% viable cells in all concentrations, Figure 1A ). The camptothecin (positive control) significantly reduced viability (54% viable cells). The MTS indicated significant cytotoxicity of POH at 250 µM ( Figure 1B ).

The POH effect (10-250 µM) on proliferation was determined by direct cell counting at 24, 48, and 72 h. The POH at 10-250 µM significantly inhibited proliferation of macrophages only at 72 h; however, the POH at 100 µM significantly reduced the total number of cells from 48 h ( Figure 1C ). Two non-cytotoxic concentrations of POH (10 and 100 µM), as determined in these proliferation and cytotoxicity assays, were used in the subsequent experiments.

### ROS production

**Figure 2:**
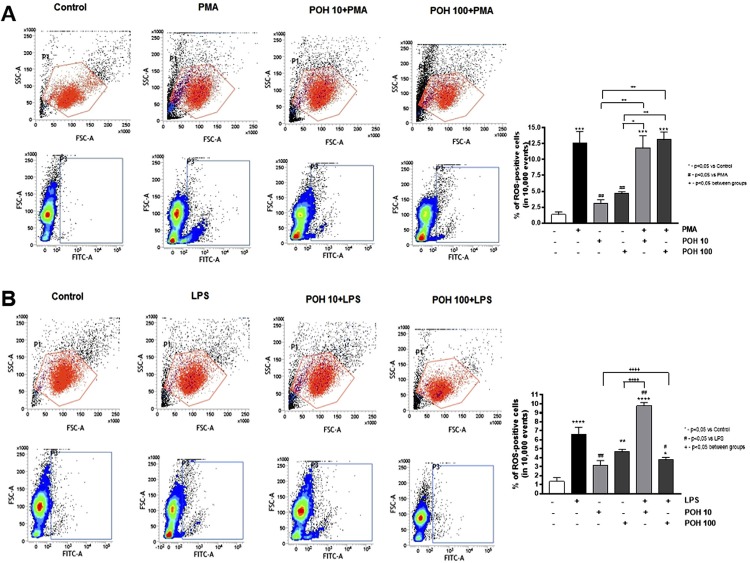
POH effect on ROS production induced by PMA (A) and LPS (B) in RAW 264.7 macrophages. The cells were treated with 10 µM and 100 µM of POH for 30 minutes, followed by a 20-minute stimulation with 50 ng/mL PMA or 10 µg/mL LPS. The ROS production was assessed by flow cytometry by measuring the oxidation of the substrate 6-carboxy-2',7'-dichlorodihydrofluorescein diacetate, which emits fluorescence in the green channel (FITC) upon excitation at 488 nm. Representative dot-plots of flow cytometry analysis depicting the percentage of ROS-positive (FITC-positive) cells. Significant differences are based on the ANOVA with a *post-hoc* Tukey test. * p<0.05, ** p<0.01, *** p<0.001 versus control or as indicated in the figure

The POH alone at 100µM caused a statistically significant increase in the ROS production when compared to the control group (p value <0.01). However, it did not affect the PMA-induced production of ROS at either concentration (10 µM and 100 µM) ( Figure 2A ). Conversely, the POH had a concentration-dependent effect on the LPS-induced ROS production. At 10 µM, the POH had a significant (p<0.01) synergistic effect with the LPS on ROS induction, whereas at 100 µM there was a significant reduction in the LPS-induced ROS production (p<0.05) ( Figure 2B ).

### Gene expression of TNF and Arginase 1

**Figure 3:**
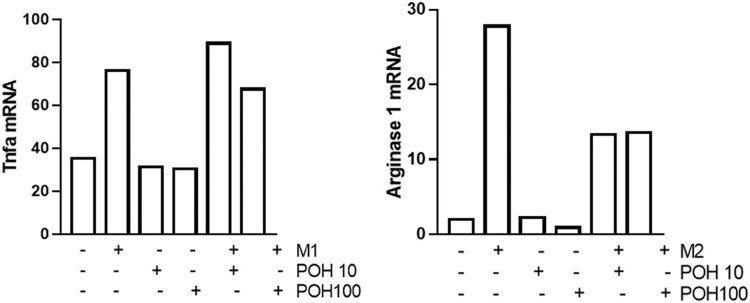
Expression of TNF and Arginase-1 assessed by the RT-qPCR in RAW 264.7 cells. M1 stimuli were *E. coli* LPS (1 μg/mL) and IFNγ (100 ng/mL) in pre-treated cells (30 min) with 10 µM or 100 µM of POH (control cells were treated with the same vehicle of the vehicle used to dilute POH). Columns represent the means for two independent experiments

The POH alone (10 µM and 100 µM) did not affect expression of TNF or Arginase-1 by macrophages. The TNF was increased by stimulation with LPS and IFN-gamma, and pre-treatment with POH (10 and 100 µM) had no effect. The POH partially inhibited IL-4-induced expression of Arginase-1 POH at both 10 and 100 µM ( Figure 3 ).

## Discussion

POH is a natural monoterpene found in several essential oils, and its antitumor activity has been well established in the literature and supported by *in vitro* and *in vivo* studies, and Phase II clinical trials in cancer patients.^10 - 12 , 15 , 27 , 28^ Recent studies have suggested that the POH also plays a role in the immunoinflammatory response.^18 , 19^

Periodontitis is a chronic inflammatory condition associated with a dysbiotic microbial biofilm that may benefit from immuno-regulatory properties of the POH. The researchers of this study did not find any studies on the antibacterial activity of the POH against periodontal pathogens. The POH had MIC and MBC values of 1600 μM (250 μg/mL) for two prominent Gram-negative microorganisms associated with periodontal disease: *P. gingivalis* and *F. nucleatum* , with bactericidal action. Natural products with MIC values between 101 and 500 μg/mL are considered strong inhibitors of microbial activity.^29^ These results will be expanded to include other Gram-negative species associated with periodontal diseases, such as *Aggregatibacter actinomycetemcomitans.* Another data limitation of the study is the assessment of the antibacterial activity in the planktonic form as opposed to the biofilm assays.

POH was not cytotoxic to human peripheral blood mononuclear cells (PBMCs) at 0.625-5 mM.^30^ Specifically in RAW264.7 cells, the reported IC_50_ of POH diluted in 10% DMSO was above 50 µM.^31^ Agreeing with these data, this study showed that the POH was not cytotoxic to RAW264.7 cells up to a concentration of 100 µM. All subsequent experiments used non-cytotoxic concentrations of POH to avoid possible bias.

The literature reports contrasting POH effects on cell proliferation. Different experimental conditions, such as POH concentrations and cell types may account for the discrepancies. Toro-Arreola, et al.^32^ (2005) observed that 0.025 µM POH was a weak inducer of proliferation of murine lymphocytes, whereas higher POH concentrations inhibited proliferation. In human peripheral blood lymphocytes, the POH had a concentration-dependent (0.625-5 mM) inhibitory effect on proliferation.^30^ The POH significantly reduced proliferation of murine macrophages *in vitro* starting at 48 h of treatment (100 µM), with a more pronounced effect at 72 h (100 µM and 250 µM).

ROS production is a major biological process in stimulated macrophages involved in the killing of phagocytized microorganisms and in the production of other soluble inflammatory mediators.^33^ The POH has been shown to have both pro- and antioxidant effects, especially in tumor cells.^34 , 35^ However, there are no studies assessing the POH effect on the ROS production to compare with macrophages. In this study, the POH alone at 100 µM induced a statistically significant increase in the ROS production and did not affect ROS production induced by PMA. Interestingly, the POH effect on the LPS-induced ROS was concentration-dependent: 10 µM further increased and 100 µM reduced LPS-induced ROS. This suggests that the POH modulates ROS production induced via activation of TLR4,^36^ but not via the protein kinase Cα (PKCα) pathway activated by PMA.^37^

An important characteristic of macrophages is their phenotypic plasticity in response to the microenvironmental cues. This study assessed the expression of TNF and Arginase-1 as genes representative of the M1/pro-inflammatory and M2/reparative phenotypes, respectively. The POH alone had no effect on the expression of TNF or Arginase-1. Moreover, the TNF expression induced by M1-polarizing stimuli was also not affected by POH. Interestingly, the POH at 10 and 100 µM reduced the expression of Arginase-1 induced by M2-polarizing stimulus. These results suggest that POH may favor the M1 phenotype, although it is important to note the limitation of assessing gene expression and not protein production.

Macrophage polarization is a complex process finely controlled by intracellular signaling pathways activated by the external stimuli. The M1-inducing stimuli used in this study activates predominantly STAT1 (IFNγ) and MAPKs and NF-kB.^38^ The IL-4 used as an M2-polarizing stimulus activates primarily STAT6.^39^ The reduction of IL-4-induced Arginase-1 by POH suggests an inhibition of STAT6, as inhibition of STAT6 by siRNA or the biochemical inhibitor AS1517499 inhibits expression of Arginase-1 and Arginase activity induced by IL-4 and IL-13 in RAW264.7 cells.^40^ It is intriguing that the POH modulates the TLR-induced ROS, which also involves MAPK and NF-kB as major downstream signaling pathways, but not the TNF expression. These possibilities should be assessed in future experiments, which should also assess the POH effect on periodontitis and the associated inflammation *in vivo* .

## Conclusion

POH has a strong antibacterial effect against *P. gingivalis.* and *F. nucleatum.* Up to 100 µM, the POH was not cytotoxic to murine macrophages, but reduced proliferation, LPS-induced ROS production, and IL-4-induced expression of Arginase-1.

## References

[1] - Dentino A, Lee S, Mailhot J, Hefti AF. Principles of periodontology. Periodontol 2000. 2013;61(1):16-53. doi: 10.1111/j.1600-0757.2011.00397.x 10.1111/j.1600-0757.2011.00397.x 23240942

[2] - Hassel TM. Tissues and cells of the periodontium. Periodontol 2000. 1993;3(1):9-38. doi: 10.1111/j.1600-0757.1993.tb00230.x 10.1111/j.1600-0757.1993.tb00230.x 9673156

[3] - Holden JA, Attard TJ, Laughton KM, Mansell A, O’Brien-Simpson NM, Reynolds EC. Porphyromonas gingivalis lipopolysaccharide weakly activates M1 and M2 polarized mouse macrophages but induces inflammatory cytokines. Infect Immun. 2014;82(10):4190-203. doi: 10.1128/IAI.02325-14 10.1128/IAI.02325-14 PMC4187848 25047849

[4] - Lam RS, O’Brien-Simpson NM, Holden JA, Lenzo JC, Fong SB, Reynolds EC. Unprimed, M1 and M2 macrophages differentially interact with Porphyromonas gingivalis. PLoS ONE. 2016;11(7):1-17. doi: 10.1371/journal.pone.0158629 10.1371/journal.pone.0158629 PMC4934774 27383471

[5] - Kwan Tat S, Padrines M, Théoleyre S, Heymann D, Fortun Y. IL-6, RANKL, TNF-alpha/IL-1: interrelations in bone resorption pathophysiology. Cytokine Growth Factor Rev. 2004;15(1):49-60. doi: 10.1016/j.cytogfr.2003.10.005 10.1016/j.cytogfr.2003.10.005 14746813

[6] - Chávez-Galán L, Olleros ML, Vesin D, Garcia I. Much more than M1 and M2 macrophages, there are also CD169(+) and TCR(+) macrophages. Front Immunol. 2015;6:1-15. doi: 10.3389/fimmu.2015.00263 10.3389/fimmu.2015.00263 PMC4443739 26074923

[7] - Guimarães MR, Coimbra LS, Aquino SG, Spolidorio LC, Kirkwood KL, Rossa C Jr. Potent anti-inflammatory effects of systemically administered curcumin modulate periodontal disease in vivo. J Periodontal Res. 2011;46(2):269-79. doi: 10.1111/j.1600-0765.2010.01342.x 10.1111/j.1600-0765.2010.01342.x PMC3086370 21306385

[8] - Jayash SN, Hashim NM, Misran M, Baharuddin NA. In vitro evaluation of osteoprotegerin in chitosan for potential bone defect applications. PeerJ. 2016;4:e2229. doi: 10.7717/peerj.2229 10.7717/peerj.2229 PMC5012333 27635307

[9] - Shojaei S, Kiumarsi A, Moghadam AR, Alizadeh J, Marzban H, Ghavami S. Perillyl alcohol (monoterpene alcohol), limonene. Enzymes. 2014;36(7):7-32. doi: 10.1016/B978-0-12-802215-3.00002-1 10.1016/B978-0-12-802215-3.00002-1 27102697

[10] - Belanger JT. Perillyl alcohol: applications in oncology. Altern Med Rev. 1998;3(6):448-57. 9855569

[11] - Kelloff GJ, Boone CW, Crowell JA, Steele VE, Lubet RA, Doody LA, et al. New agents for cancer chemoprevention. J Cell Biochem. 1996;26:1-28. doi: 10.1002/jcb.240630703 10.1002/jcb.240630703 9154166

[12] - Tyler VE, Brady LR, Robbers JE. Pharmacognosy. 9th ed. Philadelphia: Lea & Febiger; 1988.

[13] - Chen TC, Fonseca CO, Schönthal AH. Preclinical development and clinical use of perillyl alcohol for chemoprevention and cancer therapy. Am J Cancer Res. 2015;5(5):1580-93. PMC4497427 26175929

[14] - Elegbede JA, Flores R, Wang RC. Perillyl alcohol and perillaldehyde induced cell cycle arrest and cell death in BroTo and A549 cells cultured in vitro. Life Sci. 2003;73(22):2831-40. doi: 10.1016/s0024-3205(03)00701-x 10.1016/s0024-3205(03)00701-x 14511768

[15] - Gómez-Contreras PC, Hernández-Flores G, Ortiz-Lazareno PC, Toro-Arreola S, Delgado-Rizo V, Lerma-Díaz, et al. In vitro induction of apoptosis in U937 cells by perillyl alcohol with sensitization by pentoxifylline: increased BCL-2 and BAX protein expression. Chemotherapy. 2016;52(6):308-15. doi: 10.1159/000096003 10.1159/000096003 17008791

[16] - Grassmann J. Terpenoids as plant antioxidants. Vitam Horm. 2005;72:505-35. doi: 10.1016/S0083-6729(05)72015-X 10.1016/S0083-6729(05)72015-X 16492481

[17] - Stayrook KR, McKinzie JH, Burke YA, Crowell PL. Induction of the apoptosis-promoting protein Bak by perillyl alcohol in pancreatic ductal adenocarcinoma relative to untransformed ductal epitelial cells. Carcinogenesis. 1997;18(8):1655-8. doi: 10.1093/carcin/18.8.1655 10.1093/carcin/18.8.1655 9276644

[18] - D’Alessio PA, Mirshahi M, Bisson JF, Bene MC. Skin repair properties of d-Limonene and perillyl alcohol in murine models. AntiInflamm Antiallergy Agents Med Chem. 2014;13(1):29-35. doi: 10.2174/18715230113126660021 10.2174/18715230113126660021 24160248

[19] - Khan AQ, Nafees S, Sultana S. Perillyl alcohol protects against ethanol induced acute liver injury in Wistar rats by inhibiting oxidative stress, NFk-B activation and proinflammatory cytokine production. Toxicology. 2011;279:108-14. doi: 10.1016/j.tox.2010.09.017 10.1016/j.tox.2010.09.017 20923693

[20] - Tomaz-Morais JF, Braga RM, Sousa FB, Sousa DP, Pordeus LC, Almeida RN, et al. Orofacial antinociceptive activity of (S)-(-)-perillyl alcohol in mice: a randomized, controlled and triple-blind study. Int J Oral Maxillofac Surg. 2017;46(5):662-7. doi: 10.1016/j.ijom.2017.01.024 10.1016/j.ijom.2017.01.024 28233648

[21] - Clinical and Laboratory Standards Institute - CLSI. M11-A5: Methods for antimicrobial susceptibility testing of anaerobic bacteria. 7th edition. Clinical and Laboratory Standards Institute, Wayne: Clinical and Laboratory Standards Institute: 2007.

[22] - Matsebatlela TM, Anderson AL, Gallicchio VS, Elford H, Rice CD. 3,4-Dihydroxy-benzohydroxamic acid (Didox) suppresses pro-inflammatory profiles and oxidative stress in TLR4-activated RAW264.7 murine macrophages. Chem Biol Interact. 2015;233:95-105. doi: 10.1016/j.cbi.2015.03.027 10.1016/j.cbi.2015.03.027 PMC4408267 25843059

[23] - Woo CH, Lim JH, Kim JH. Lipopolysaccharide induces matrix metalloproteinase-9 expression via a mitochondrial reactive oxygen species-p38 kinase-activator protein-1 pathway in Raw 264.7 cells. J Immunol. 2004;173(11):6973-80. doi: 10.4049/jimmunol.173.11.6973 10.4049/jimmunol.173.11.6973 15557194

[24] - Yuan X, Cao H, Wang J, Tang K, Li B, Zhao Y, et al. Immunomodulatory effects of calcium and strontium co-doped titanium oxides on osteogenesis. Front Immunol. 2017;8:1196. doi: 10.3389/fimmu.2017.01196 10.3389/fimmu.2017.01196 PMC5626827 29033930

[25] - Berridge MV, Tan AS. Characterization of the cellular reduction of 3-(4,5-dimethylthiazol-2-yl)-2,5-diphenyltetrazolium bromide (MTT): subcellular localization, substrate dependence, and involvement of mitochondrial electron transport in MTT reduction. Arch Biochem Biophys. 1993;303(2):474-82. doi: 10.1006/abbi.1993.1311 10.1006/abbi.1993.1311 8390225

[26] - Cory AH, Owen TC, Barltrop JA, Cory JG. Use of an aqueous soluble tetrazolium/formazan assay for cell growth assays in culture. Cancer Commun. 1991;3(7):207-12. doi: 10.3727/095535491820873191 10.3727/095535491820873191 1867954

[27] - Bailey HH, Attia S, Love RR, Fass T, Chappell R., Tutsch K, et al. Phase II trial of daily oral perillyl alcohol (NSC 641066) in treatment-refractory metastatic breast cancer. Cancer Chemother Pharmacol. 2008;62(1):149-57. 10.1007/s00280-007-0585-6 10.1007/s00280-007-0585-6 17885756

[28] - Chaudhary SC, Alam MS, Siddiqui MS, Athar M. Perillyl alcohol attenuates Ras-ERK signaling to inhibit murine skin inflammation and tumorigenesis. Chem Biol Interact. 2009;179(2-3):145-53. doi: 10.1016/j.cbi.2008.12.016 10.1016/j.cbi.2008.12.016 19161993

[29] - Freires IA, Denny C, Benso B, Alencar SM, Rosalen PL. Antibacterial activity of essential oils and their isolated constituents against cariogenic bacteria: a systematic review. Molecules. 2015;20(4):7329-59. doi: 10.3390/molecules20047329 10.3390/molecules20047329 PMC6272492 25911964

[30] - Schulz S, Bühling F, Ansorge S. Prenylated proteins and lymphocyte proliferation: inhibition by d-limonene and related monoterpenes. Eur J Immunol. 1994;24(2):301-7. doi: 10.1002/eji.1830240204 10.1002/eji.1830240204 8299679

[31] - Gerhäuser G, Klimo K, Heiss E, Neumann I, Gamal-Eldeen A, Knauft J, et al. Mechanism-based in vitro screening of potential cancer chemopreventive agents. Mutat Res. 2003;523–524:163-72. doi: 10.1016/s0027-5107(02)00332-9 10.1016/s0027-5107(02)00332-9 12628514

[32] - Del Toro-Arreola S, Flores-Torales E, Torres-Lozano C, Del Toro-Areola A, Tostado-Pelayo K, Ramirez-Dueñas MG, et al. Effect of D-limonene on imune response in BALB/c mice with lymphoma. Int Immunopharmacol. 2005;5(5):829-38. doi: 10.1016/j.intimp.2004.12.012 10.1016/j.intimp.2004.12.012 15778119

[33] - Dupré-Crochet S, Erard M, Nüße O. ROS production in phagocytes: why, when, and where? J Leukoc Biol. 2013;94(4):657-70. doi: 10.1189/jlb.1012544 10.1189/jlb.1012544 23610146

[34] - Gomes AC, Mello AL, Ribeiro MG, Garcia DG, Fonseca CO, Salaza MD, et al. Perillyl alcohol, a pleiotropic natural compound suitable for brain tumor therapy, targets free radicals. Arch Immunol Ther Exp. 2017;65(4):285-97. doi: 10.1007/s00005-017-0459-5 10.1007/s00005-017-0459-5 28314870

[35] - Xu Y, Wang W, Jin K, Zhu Q, Lin H, Xie M, et al. Perillyl alcohol protects human renal tubular epithelial cells from hypoxia/reoxygenation injury via inhibition of ROS, endoplasmic reticulum stress and activation of PI3K/Akt/eNOS pathway. Biomed Pharmacother. 2017;95:662-9. doi: 10.1016/j.biopha.2017.08.129 10.1016/j.biopha.2017.08.129 28886525

[36] - Takeda K, Kaisho T, Akira S. Toll-like receptors. Annu Rev Immunol. 2003;21:335-76. doi: 10.1146/annurev.immunol.21.120601.141126 10.1146/annurev.immunol.21.120601.141126 12524386

[37] - Kohl R, Preiss S, von Knethen A, Brüne B. Oxidized low-density lipoprotein depletes PKCalpha and attenuates reactive oxygen species formation in monocytes/macrophages. Cardiovasc Res. 2006;71(3):574-85. doi: 10.1016/j.cardiores.2006.05.023 10.1016/j.cardiores.2006.05.023 16843450

[38] - Wang N, Liang H, Zen K. Molecular mechanisms that influence the macrophage m1-m2 polarization balance. Front Immunol. 2014;5:614. doi: 10.3389/fimmu.2014.00614 10.3389/fimmu.2014.00614 PMC4246889 25506346

[39] - Han MS, Jung DY, Morel C, Lakhani SA, Kim JK, Flavell RA, et al. JNK expression by macrophages promotes obesity-induced insulin resistance and inflammation. Science. 2013;339(6116):218-22. doi: 10.1126/science.1227568 10.1126/science.1227568 PMC3835653 23223452

[40] - Binnemars-Postma K, Bansal R, Storm G, Prakash J. Targeting the Stat6 pathway in tumor-associated macrophages reduces tumor growth and metastatic niche formation in breast cancer. FASEB J. 2018;32(2):969-78. doi: 10.1096/fj.201700629R 10.1096/fj.201700629R 29066614

